# Recent Advances in UV-Cured Encapsulation for Stable and Durable Perovskite Solar Cell Devices

**DOI:** 10.3390/polym15193911

**Published:** 2023-09-28

**Authors:** Mengyu Cao, Wenxi Ji, Cong Chao, Ji Li, Fei Dai, Xianfeng Fan

**Affiliations:** 1 SINOPEC (Beijing) Research Institute of Chemical Industry Co., Ltd., Beijing 100013, China; caomy.bjhy@sinopec.com (M.C.); jiwx.bjhy@sinopec.com (W.J.); liji.bjhy@sinopec.com (J.L.); 2 Beijing Key Laboratory of Emission Surveillance and Control for Thermal Power Generation, North China Electric Power University, Beijing 102206, China; 50202852@necpu.edu.cn; 3 Laboratory of Distributed Energy System and Renewable Energy, Institute of Engineering Thermophysics, Chinese Academy of Sciences, Beijing 100190, China; 4 Institute for Materials and Processes, School of Engineering, The University of Edinburgh, Edinburgh EH9 3FB, UK

**Keywords:** encapsulation, perovskite solar cells, UV-cured resin

## Abstract

The stability and durability of perovskite solar cells (PSCs) are two main challenges retarding their industrial commercialization. The encapsulation of PSCs is a critical process that improves the stability of PSC devices for practical applications, and intrinsic stability improvement relies on materials optimization. Among all encapsulation materials, UV-curable resins are promising materials for PSC encapsulation due to their short curing time, low shrinkage, and good adhesion to various substrates. In this review, the requirements for PSC encapsulation materials and the advantages of UV-curable resins are firstly critically assessed based on a discussion of the PSC degradation mechanism. Recent advances in improving the encapsulation performance are reviewed from the perspectives of molecular modification, encapsulation materials, and corresponding architecture design while highlighting excellent representative works. Finally, the concluding remarks summarize promising research directions and remaining challenges for the use of UV-curable resins in encapsulation. Potential solutions to current challenges are proposed to inspire future work devoted to transitioning PSCs from the lab to practical application.

## 1. Introduction

Perovskite solar cells (PSCs) present significant advantages as a promising photovoltaic (PV) cell for the future. In the last decade, PSCs have achieved a significant improvement of ~670% in power conversion efficiency (PCE). To date, the highest certificated PCE of PSC is 26% for single-junction cells and 33.7% for perovskite/Si tandem cells, respectively [1]. The PCE of perovskite-Si stacking tandem cells has surpassed the traditional two-junction PV record without a sun concentrator on the NREL Best Research-Cell Efficiency Chart [2,3,4]. In addition to the ongoing PCE improvements, the commercial attractiveness of PSCs also derives from their economic advantages: they can be manufactured using cost-effective materials and solution-based processes, in contrast with the much more expensive, over 1000 °C temperature, and high-purity processes required by traditional silicon-based solar cells. Moreover, PSCs are characterized by their lightweight and flexibility, which permits their integration into curved or irregular surfaces including building-integrated photovoltaics and portable and wearable electronics. However, technoeconomic analyses suggest that the economic advantages of PSC compared with commercial Si PV can only be realized when desirable performance and lifetime are reached. For now, the cost of the PSC module, which largely determines its market potential for replacing traditional Si-based PV, is more significantly hindered by the lifetime than the performance of its PSC, considering that traditional Si-based PV lasts for at least 15 years [5,6,7]. Addressing the challenge of enhancing the lifespan of PSCs is considered a top priority by both academia and industry, as it is crucial for unlocking their full potential in practical applications. 

The fundamental issue of PSC stability derives from the unique organic–inorganic hybrid properties of the perovskite absorption layer, which is sensitive to external factors such as moisture, oxygen, UV irradiation, and heat during processing and practical applications. The strategies used to address the lifetime issue of PSCs can be classified into two categories: (1) increasing the stability of perovskite crystals from the perspective of materials design and (2) providing an inert environment with durable encapsulation structure and materials acting as barriers to the oxygen and water in the environment. Compared with stability improvements relying on materials design [8,9], important encapsulation materials and structures are much less systematically investigated and understood. The encapsulation of PSCs is critical for industry because PSC cells must be brought from the laboratory to the harsh practical application environment, and the encapsulation method needs to be carefully evaluated based on large-scale production cost as well as scaling-up possibilities. Encapsulation methods can be generally categorized into (1) external encapsulation, mainly including UV-cured adhesive encapsulation within glass coverslips [10] and (2) internal techniques of grain boundary encapsulation [11] and interface passivation [12]. Compared with internal encapsulation techniques, external encapsulation methods generally offer a stronger barrier effect against the environment [13]. In the literature, many recent works aim to develop UV-cured encapsulation resins and their suitable encapsulation structures integrated into PSC cells, which have proven effective in enhancing the lifetime and endurability of PSCs. However, PSC stability is still far from satisfactory, which requires further enhancing the barrier performance of UV-cured encapsulation resins and achieving better compatibility with PSCs. To date, the literature lacks a holistic review of the UV-cured encapsulation that summarizes the recent advances and performs a systematic assessment. An understanding of the working principle and mechanisms underlying UV-cured encapsulation for enhancing the long-term stability of PSCs is severely needed. 

Focusing on the encapsulation strategy to enhance PSC stability, this review summarizes recent advances in the development of encapsulation resins as well as their deployment in real devices. The PSC degradation mechanism is first briefly summarized and, accordingly, the requirements for practical cell encapsulation resins are discussed from the perspectives of photoinitiator design, water permeability enhancement, strategies for resin toughening, and mechanical property improvement. The applications of encapsulation resins on PSCs and their performances in enhancing stability are reviewed. Recent important advances are highlighted to demonstrate the effectiveness of UV-cured resins in promoting the long-term stability of PSCs. A summary and concluding remarks are presented at the end of this review followed by an outlook on the future developments for UV-cured PSC encapsulations.

## 2. Materials and Methods Used for PSC Encapsulation

### 2.1. The PSC Degradation Mechanism and the Requirements for PSC Encapsulation Materials

The mechanisms underlying the degradation of PSC devices can be categorized by internal and external factors: the internal instability of perovskite is related to its defects, crystal structure, and thermal instability; and the external instability factors include UV irradiation, temperature, moisture, and oxygen, which need to be prevented using the encapsulation [14,15]. To be specific, perovskite crystals are normally susceptible to moisture and oxygen; exposure under illumination accelerates the degradation of PSC devices with the presence of moisture and oxygen. The most commonly reported degradation mechanisms are summarized as follows. (1) When both moisture and oxygen are present, they very easily generate strong hydrogen bonds with perovskite organic cations to form a hydrated intermediate. The formation of an intermediate product will decrease absorption within the visible region and change the crystal structure [16,17,18]. Due to the weak chemical bond between the organic cation and the intermediate framework, the organic cation is more likely to escape from the bulk of perovskite, and faster deprotonation of the organic cation will accelerate the degradation of PSC devices. In addition, volatile hydrohalogenic acid is formed after the protonation of perovskite with water, and perovskite materials can be degraded into metal halide after a few hundred hours [19]. (2) Oxygen is prone to interacting with vacancies in perovskite, which accelerates its degradation. When oxygen diffuses into the bulk of the perovskite absorber, it can easily occupy the anion vacancies and trap the electrons in the perovskite films [20]. Then, the formed reactive superoxide (O_2_^−^) will trigger an acid–base reaction to degrade perovskite into water, deprotonated A-site gas, and metal halogenides. (3) It has been reported that light irradiation significantly accelerates the degradation of CTLs (charge transport layers) of PSC devices. Light irradiation causes structural changes in perovskite crystals such as vacancy generation, phase segregation, ion migration, and compositional degradation [21,22,23,24]. For example, upon UV light exposure, meso-TiO_2_ may react with adsorbed oxygen molecules to generate complex O_2_^−^Ti^4+^ with the presence of oxygen vacancies or Ti^3+^ states at the surface of TiO_2_ [25]. (4) The influence of heat on PSCs also needs to be considered, as solar cells are inevitably exposed to thermal treatments during fabrication and operation processes. In addition, thermo-induced phase decomposition, operating at elevated temperatures, has also been reported to accelerate the performance deterioration of PSCs in practical applications by activating ion migration, causing undesirable aggregation, or chemical reactions in perovskite films [26,27,28]. For instance, Kim et al. reported that MAPbI_3_ perovskite films were decomposed into CH_3_I, NH_3_, and PbI_2_ after a short exposure (about 20 min) to the temperature of 100 °C [29]. Except for perovskite films, thermal degradation may also occur in other functional layers of PSCs, such as spiro-OMeTAD (2,2′7,7′-tetrakis (N,N-di-p-methoxyphenylamine)-9,9′-spirobifluorene) and metal-involved electrode materials [30]. The open-circuit voltage (Voc) of a PSC device decreases with an increase in temperature, and the current density (Jsc) of PSCs is also found to decrease to 20% of the initial value when the test temperature cycles from 10 to 60 °C. These observations all indicate the performance decline in PSCs caused by thermal treatment [31,32].

Based on the understanding of the PSC degradation mechanism, ideal encapsulation materials are expected to possess excellent barrier properties against moisture, oxygen, and low processing temperatures. As part of the encapsulation, the materials should also show desirable mechanical properties such as low elastic modulus, minimal residual mechanical stress, appropriate thermal expansion coefficient, and anti-creep behaviour. These desired properties primarily aim at withstanding temperature cycling and preventing delamination during thermal cycling testing. From a chemical perspective, the encapsulation material should exhibit high stability and strong adhesive properties. Additionally, the materials should fulfil the general requirements of PV encapsulation materials such as high surface and volume resistivity, significant optical transmittance, and facility for large-scale processing [33,34,35,36,37,38]. All these factors collectively contribute to the overall performance of encapsulation in practical applications. To date, there are no generally accepted specifications for PSC encapsulation materials. Similar to PSCs, the active layer and transport layer of organic photovoltaic (OPV) cells are also sensitive to water vapor and oxygen molecules, making the encapsulation material specifications for PSCs applicable to OPV cells as well. The specification list for OPV encapsulation materials is usually used as a good reference (Table 1) [30,35,39]. However, it is important to note that PSCs generally exhibit lower thermal stability compared with OPV cells, which implies that stricter temperature requirements should be imposed on encapsulation materials for PSCs [40]. For performance evaluation, the International Summit on Organic Photovoltaic Stability (ISOS) protocols [41] were proposed and are generally accepted in the research community.

### 2.2. Comparison of Different Encapsulation Structures, Methods, and Materials

**Figure 1:**
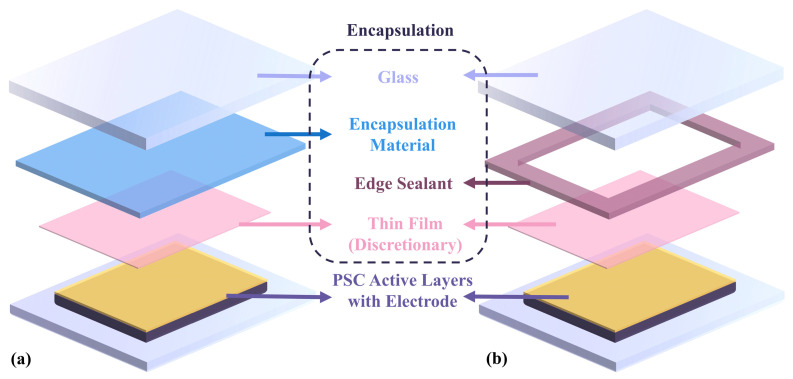
Side view of typical encapsulation: (**a**) cover-seal and (**b**) edge-seal structure of PSCs.

Si PV encapsulation techniques are well-documented and mature in the industry. However, for emerging PSCs with sensitive crystal/chemical structures, most of the encapsulation methods and materials need to be thoroughly modified to adapt to the specific requirements of PSCs. The encapsulation structure usually contains a covering layer and encapsulant, which can be generally categorized into two kinds: cover-sealed structure and side-sealed structure. As shown in Figure 1a, in the cover-sealed structure, glass or plastic coverslips are used to cover PSCs and provide a barrier against moisture and mechanical damage. This structure, which originated from silicon solar cells, utilizes coverslips in conjunction with other encapsulation materials [42,43] to provide complete protection for the perovskite core layer. A scheme showing an edge-sealed encapsulation structure is provided in Figure 1b, where the whole PSC unit between coverslips and ITO is sealed with edge sealants. The edge-sealed structure has been widely used in laboratory experiments and has proven successful in degradation alleviation [44,45]. Prior to the addition of encapsulation materials and coverslips, recent studies showed that thin-film barriers, such as Al_2_O_3_ [46], SnO_x_ [47], and TiO_2_ [48], etc., can be applied to the outer side of the perovskite core layer and metal back electrode. Furthermore, Byranvand et al. reported direct passivation of perovskite thin-film surfaces achieved using chemical vapour deposition of a polymer layer. PSCs with a ~1 nm poly(p-xylylene) passivation layer exhibited an enhanced open-circuit voltage (V_OC_) and fill factor and demonstrated protective capabilities under high humidity conditions (relative humidity ~40–50%) [49]. The additional functional thin film can effectively improve the stability of perovskite solar cells with different functions.

Generally, two methods have been used in the literature to encapsulate PSCs: glass-glass laminated encapsulation and UV-cured encapsulation. Glass–glass laminated encapsulation is usually applied with thermosetting polymers. EVA (ethylene vinyl acetate) film is one of the most commonly used encapsulation materials for glass–glass laminated encapsulating [10,50]. However, EVA was found to be highly water-absorbing [51,52] and undergoes slow UV light-driven hydrolysis, which produces acetic acid and corrodes the perovskite core layer [53]. The POE (polyolefin elastomer) and TPO (thermoplastic olefin) films offer notable performance and chemical inertness, making them promising encapsulant candidates for PSCs [54,55,56]. Unfortunately, high processing temperatures and high costs limit their widespread usage [57,58,59]. In addition to the previously mentioned film materials, other encapsulation film materials including PIB (polyisobutylene) [60,61], PET (polyethylene) [62], PMMA (poly (methyl methacrylate) [63], EVOH (ethylene-vinyl alcohol copolymer) [64], and thermoplastic polyurethane (TPU) [65,66] have also been reported in the literature. The long-term stability of encapsulants tested in the real environment is invaluable for assessing their performance. For example, polyurethane in four different OPV cell configurations was tested across eight countries for 4.5 months. The results suggested that the OPV cell preserved around 40% of the original efficiency and that photodegradation is not the main reason for the decreased efficiency [67].

However, the biggest challenge with thermoplastic encapsulation films is their high lamination temperature (usually higher than 140 °C [10]). For example, EVA film requires ~20 min vacuum lamination at around 150 °C, which is much higher than the thermal tolerance temperature of PSCs (for example, MAPbI_3_ perovskite material starts to decompose at ~85 °C [29]). The UV-cured encapsulation method using UV-curable resins can be conducted at room temperature (RT) within minutes under UV illumination, which provides the UV-cured encapsulation method with significant advantages [56,66,68]. In comparison with the glass–glass laminated encapsulation method, UV-cured encapsulation minimizes the adverse effects of high temperature on the perovskite core layer [69,70]. Matteocci et al. [71] investigated devices with Surlyn^TM^ 60 film and saw a PCE reduction of 58% after 170 h. However, those with UV-curable glue resulted in only a 21% decline over a similar timeframe. During the encapsulation process, the Surlyn^TM^ 60 film was applied to the PSC with a hot press at 100 °C and 0.4 bar for 40 s, while the light-curable glue was cured under a Xenon lamp for 10 s at room temperature. In Shi et al.’s work [61], PSCs encapsulated with EVA films and laminated at 135 °C, 145 °C, and 155 °C for 15 min held less than 50% PCE post 140 h. In contrast, those sealed with UV-cured epoxy edge seals curing under UV light for 10 min at room temperature achieved a PCE close to 60%, reflecting superior J-V measurement outcomes. The authors suggested that excessively high hot-pressing temperatures are not suitable for the PSC encapsulation process. Additionally, the short curing process offers significantly improved production efficiency of PSCs, which helps to reduce the cost of the final products [72,73]. From the perspective of industrial application, UV-cured encapsulation methods are efficient for large-scale production because they energy consumption, and thus are suitable for high-speed automated production lines. Most importantly, UV-curable resins also show impressive resistance to both chemical and environmental factors. For example, Baranwal et al. [74] confirmed that the UV-curing adhesive from Three Bond Holding Co., Ltd. (Tokyo, Japan) (3035B) had the capability to block the migration of iodide and lead at the temperature of 100 °C, which is quite a high temperature for a perovskite absorber. In addition to good barrier performances, UV-curable resins also possess commendable adhesion properties. Once epoxy resin undergoes 3D network crosslinking, it exhibits high adhesive strength. The epoxy groups and hydroxyl groups on the glass surface readily engage in intermolecular interactions and hydrogen bonding [75]. This ability allows the resin to firmly bond with the PSCs and other encapsulation materials, thereby strengthening the robustness of the encapsulation layer [75]. UV-curable resins also have low shrinkage properties, which ensures a low risk of detaching from the PSC or any other encapsulation materials during the curing process, giving a more uniform and solid encapsulation layer [76]. UV epoxy has many successful applications in the commercialization of organic light-emitting diodes (OLEDs) [77,78,79].

As described in the Table 2, the water vapor transmission rate (WVTR) of film materials, especially PIB, EVOH, and PET (used as a substrate), exhibit remarkably high barriers against water vapor, which is a desirable attribute for PSCs. Rather than directly heat-pressing these films onto cells, a light-curable adhesive is used to secure them, mitigating the risk of direct heat degradation on the perovskite layer. Films like EVA and POE and materials like PIB, which require laminating heat, can be applied to the cells using commercial lamination equipment. They also possess commendable moisture barrier properties, especially PIB. As more research focuses on reducing the processing temperature of these encapsulation materials, such laminated films are showing promising potential for the future [66].

In summary, UV-curable resins offer attractive advantages as an encapsulation material for PSCs. The short curing time and good adhesion properties of UV-curable resins ensure a strong and uniform encapsulation layer that provides effective protection against environmental factors. The high chemical and environmental resistance and low shrinkage properties of UV-curable resins ensure a long lifespan for the encapsulation layer, thereby improving the durability and stability of PSCs [83]. These properties make UV-curable resins a promising material for addressing the challenges of stability and durability in PSC encapsulation [84]

## 3. The Design of UV-Curable Resins for Highly Durable PSC Devices

### 3.1. The UV-Driven Curing Process

**Figure 2:**
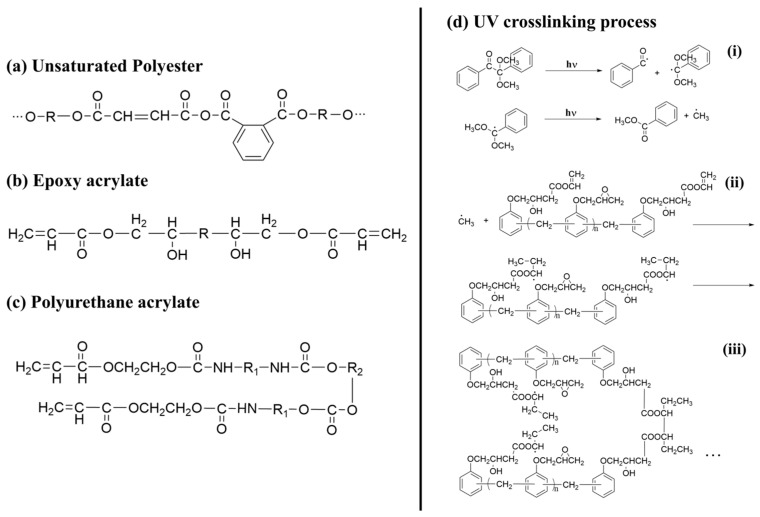
Schematic illustration showing the representative structure of (**a**) unsaturated polyester, (**b**) epoxy acrylate, (**c**) polyurethane acrylate, (**d**) the crosslinking process of UV-curable resin and free-radical type photoinitiator upon UV light irradiation, using bisphenol F epoxy resin and Irgacure 651 as examples.

**Figure 3:**
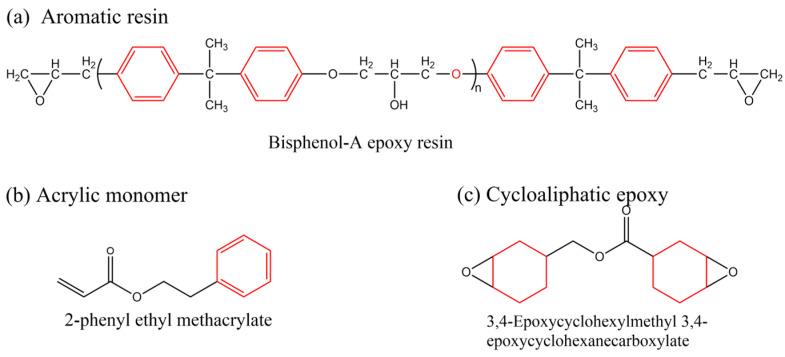
Schematics showing three distinct types of resin monomers sourced from the literature: (**a**) aromatic resin [91], (**b**) acrylic-based resin [93], and (**c**) cycloaliphatic epoxy [97].

Before beginning our discussion of UV-cured resin design for PSC encapsulation, the process of UV-curing is briefly summarized in this section. UV-cured adhesives consist of matrix resin, reactive diluents, and photo-initiators, enabling the curing process through free radical, cationic, or hybrid mechanisms [85]. Common UV-curable matrix resins include unsaturated polyesters, epoxy acrylates, polyurethane acrylates, etc., and their respective chemical structures are illustrated in Figure 2a–c. Additionally, low molecular weight epoxy resins that are commonly used as cationic photopolymers, such as aromatic resins, are depicted in Figure 3a, while the structures for cycloaliphatic epoxy resins are presented in Figure 3c. Among the various commercialized photoinitiators, benzoin and its derivatives stand out. Notably, the derivative α,α-dimethoxy-α-phenylacetophenone (DMPA), also recognized as α,α-dimethylbenzoin ketone (DMBK), is popularly marketed as Irgacure 651 [86,87]. Other key photoinitiators comprise photo-cationic types like diphenyliodonium trifluoromethanesulfonate and photo-radical variants including acetophenone. Upon exposure to UV light, the photoinitiator generates excited state molecules, which decompose into free radicals or cations and initiate chemical reactions such as polymerization and crosslinking. The UV curing process can be summarized in three steps (as shown in Figure 2d) [88,89]: (i) the excitation and decomposition of the photoinitiator (for example, Irgacure 651) using UV light, leading to the generation of free radicals and/or cations; (ii) the reaction between active free radicals (CH_3_ in Figure 2d(ii)) and the double bonds of monomers and oligomers (for example, bisphenol F epoxy resin), resulting in chain growth; and (iii) repeated reactions leading to the breaking and crosslinking of double bonds, ultimately forming a dense solid material. The graphical representation in Figure 2d illustrates this sequence of events and provides a visual understanding of the photocrosslinking mechanism. The free radical light-curing system and the cationic light-curing system are mixed to obtain a hybrid light-curing system with a good synergistic effect, such as the mixed system composed of acrylate and epoxy groups [90]. Epoxy compounds for light-curing oligomers can directly use various epoxy resins as substrates.

### 3.2. The Design of UV-Curable Resin for PSC Encapsulation

Various resin materials, such as aromatic resins [60,91,92], acrylic-based resins [68,93], cycloaliphatic epoxy resins [94,95,96], hybrid resins [97,98], etc. (structures shown in Figure 3), can be used for the encapsulation of PSC devices. After the crosslinking process, the crosslinked network structure enhances the thermal and chemical stability of the material. Furthermore, for bisphenol-A epoxy resin, the presence of the aromatic ring confers greater UV and oxidation stability. Acrylic-based resins contain an unsaturated acrylic acid functional group (red in Figure 3a), while the acrylic functional group is reactive. Upon photopolymerization or thermal curing, it forms a crosslinked structure that renders the material highly stable. Aliphatic structures in cycloaliphatic epoxy tend to be more resistant to oxidation and UV degradation than aromatic structures because they lack excitable π electrons (red in Figure 3c). Moreover, the saturated nature of this structure offers good chemical stability [99,100,101,102]. Extensive studies in the literature reported modifying monomers and additives to meet the requirements of PSC encapsulation, including water–vapor barrier properties, sensitivity to UV irradiation, low susceptibility to cracking, and excellent weather resistance. In this section, we do not limit the discussion to research specifically on PV encapsulation, instead, we broadly summarize strategies reported in the literature that enhance the crucial properties of UV-curable resins for PSC encapsulation.

#### 3.2.1. UV-Curable Resin Design to Reduce the Water Vapor Oxygen Transmission Rate

The water and oxygen instability of perovskite materials poses a significant challenge in the development of effective encapsulation strategies. WVTR (water vapor transmission rate) and OTR (oxygen transmission rate) are the most critical metrics for evaluating the effectiveness of encapsulation. The functional groups in resin are very important structural factors influencing its water/oxygen permeability. Epoxy resins containing hydroxyl groups are usually not favorable since they only form hydrogen bonds with water molecules. On the other hand, the introduction of functional groups that induce surface hydrophobicity or modify the microstructure can effectively enhance the water barrier properties of the encapsulation material.

**Figure 4:**
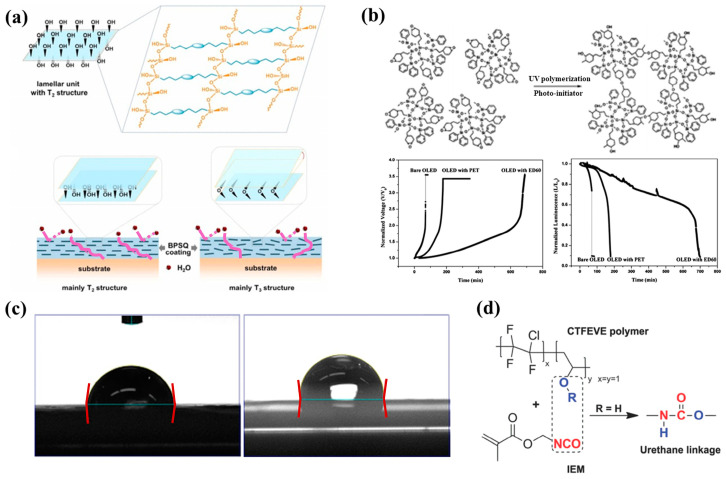
(**a**) The possible water vapor barrier mechanism of ordered and disordered lamellar nanostructures (BPSQ siloxane-based material coatings). The motion pathway of a water molecule is illustrated with the pink line. Reproduced with permission from Ref. [103]. Copyright 2022, Elsevier Ltd. (**b**) Polymerized cycloaliphatic epoxy hybrid encapsulation materials improved the stability of OLEDs. Reproduced with permission from Ref. [94]. Copyright 2015, Royal Society of Chemistry. (**c**) Fluorine polymers added to a UV-curable adhesive shows lower surface energy than the blank sample. Reproduced with permission from Ref. [105] Copyright 2015, Wiley-VCH. (**d**) Schematic representation of the mechanism underlying the formation of the photocrosslinking fluoropolymer precursor. Reproduced with permission from Ref. [106] Copyright 2014, Wiley-VCH.

For example, Zhang et al. demonstrated that polysilsesquioxane (PSQ), a siloxane-based material (Figure 4a), can effectively hinder the diffusion pathways of water molecules by introducing an oriented layered structure [103,104]. The incorporation of siloxane-based materials with enhanced water vapor barrier performance has been applied in the encapsulation of PV cells. The work by Jung et al. used a sol-gel condensation synthesis of cyclohexasiloxane epoxy hybrids using 2-(3,4-epoxycyclohexyl) ethyltrimethoxysilane (ECTS) and diphenylsilanediol (DPSD) [94]. The presence of the siloxane bond enhanced the water barrier properties of the cycloaliphatic epoxy hybrids and resulted in a visibly condensed network. This significantly improved the stability of OLEDs encapsulated with this material (as shown in Figure 4b). The WVTR of the barrier was determined using a calcium degradation method, which achieved a low value of 0.68 g m^−2^ day^−1^ per mil (25.4 μm). The method using fluorinated materials to modify UV-curable adhesives was successfully applied in dye-sensitized solar cells (DSSCs), which proved to be promising encapsulation materials for PSCs. For instance, Chiang et al. investigated the incorporation of a fluorine polymer, namely, 2-(perfluorohexyl) ethyl methacrylate (PFE), into a UV-curable adhesive [105]. Incorporating fluoropolymer in UV-curable adhesives allowed a reduction in the surface energy of the encapsulation material to approximately 13.72  ±  0.30 (mJ/m^2^), and a comparison of the contact angles can be seen in Figure 4c. This low surface energy imparts hydrophobicity to the material while also enhancing its thermal stability. Fluorinated compounds are combined with photocrosslinkable methacrylate, such as commercial chloro-tri-fluoro-ethylene vinyl ether reacted with 2-isocyanatoethyl methacrylate, to form photocrosslinkable polyurethane precursors (reaction route in Figure 4d). These fluorine-containing materials exhibit low surface energy, hydrophobicity, and thermal stability, making them suitable for applications in DSSCs [106] and PSCs [107]. In addition, the encapsulation of PSCs can be enhanced with protection by incorporating UV filtering. Yoon et al. designed a blue cut-off filter that selectively blocked blue light with wavelengths below 500 nm. This was achieved by constructing a multilayered inorganic metasurface consisting of TiO_2_ and SiO_2_ layers and applying it to a PSCs [108].

**Figure 5:**
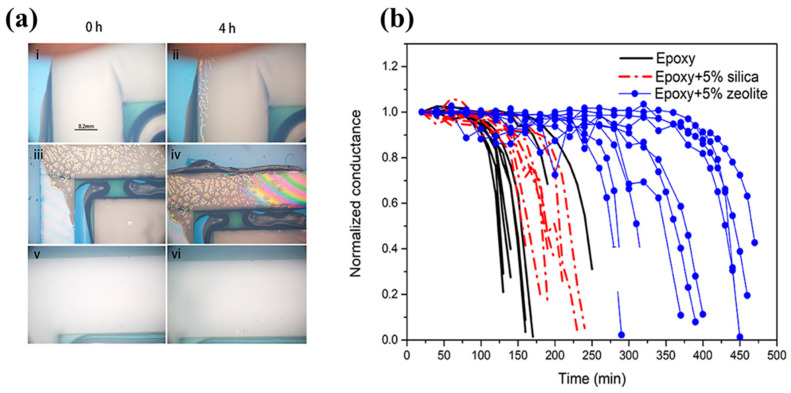
(**a**) Images (**i**,**iii**,**v**) sequentially depict Threebond UV-curable epoxy when cured between two tightly pressed glass pieces, cured without applying pressure between the cover glass and substrate, and cured with applied pressure in the presence of PIB, respectively. Conversely, images (**ii**,**iv**,**vi**) present corresponding devices after being heated at ambient temperature for 4 h, with notable bubble formation and delamination observed in the first two conditions. (**b**) Ca test for epoxy with and without incorporation of nanomaterials. Reproduced with permission under a Creative Commons CC-BY-NC-ND License from Ref. [109]. Copyright 2022 American Chemical Society.

Another approach is to combine desiccants with epoxy resins. Though the previous discussions mentioned that adding desiccants or forming voids is unfavorable for encapsulation, many researchers have suggested that a mixture of desiccants and commercial epoxy resin can be used to enhance the properties of the water vapor barrier. Easily dispersible nanomaterials are preferable since they can form a uniform blending of desiccants and resins. For example, Wang et al. [109] proposed the incorporation of zeolites, among other materials, into epoxy resin as a way to enhance the lifespan of devices. After dispersing zeolite in Threebond UV-curable epoxy, the studied device exhibited a longer lag time and lower WVTR in Ca testing, as indicated by the blue line in Figure 5b. It should be noted that the viscosity of the epoxy resin may be increased accordingly. Epoxy resins with lower viscosity are generally preferred as they have better processability and less gas entrapment, resulting in improved operability (Figure 5a) [109].

Hydroxyl-containing resins can form hydrogen bonds with water molecules. These resins used for encapsulation in liquid form may trap moisture, leading to potential degradation of the perovskite. Weerasinghe et al. [110] observed that encapsulation films with acrylic adhesive (3 M, 467 MP) exposed to environmental conditions exhibited significant moisture content. However, the moisture absorbed by encapsulation material can be effectively removed using a vacuum drying process. Therefore, to minimize the amount of moisture, it is necessary to complete the encapsulation process within a short period of time after the drying process or store the encapsulation material in a dry environment, using degassing or desiccant materials if needed.

#### 3.2.2. UV-Curable Resin Design to Retard Cracking and Aging

**Figure 6:**
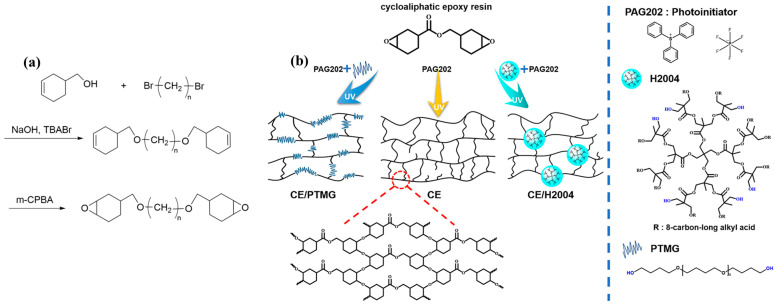
(**a**) Synthesis route of cycloaliphatic epoxy resins with -RO- segments. (**b**) Epoxy resin prepared using cationic ring-opening polymerization with PAG202 as photoinitiator and modified with hyperbranched hydroxyl polymer H2004 and linear hydroxyl polymer PTMG. Reproduced with permission from Ref. [117]. Copyright 2020, Elsevier Ltd.

It is generally believed that a too-high crosslink density of epoxy resin encapsulation usually results in brittleness and poor weather resistance, making it prone to aging and cracking under extreme weather conditions [111]. Additionally, stress generated during the curing process, combined with the material’s coefficient of thermal expansion, can lead to cracking and failure of the encapsulation layer [10,35]. On the other hand, a lower degree of crosslinking could result in a weaker adherence between the cell and the glass backing, failing to provide the desired protection. To address this challenge, toughening as a classic approach, has been applied in PSC encapsulation materials. Heterogeneous toughening methods, such as incorporating rubber, polymers, or nanomaterials, involve combining epoxy phases with toughening phases to achieve desired effects [112]. However, the use of inorganic fillers for toughening is not preferred for encapsulation resin materials in photovoltaic systems, as it would compromise the light transmittance [113]. The non-miscibility between the matrix and modifiers results in high viscosity, opacity, and low flowability, limiting the advanced processing of PV cell encapsulation. Therefore, toughening effects need to be tailored specifically for PV cells, especially for PSCs. Recently, it was found that flexible chain segments [114] and hyperbranched polymers (HBPs) [115] serve effectively on toughening modifiers, forming a well-integrated encapsulation system. For example, Crivello et al. [116] synthesized epoxy resins with cycloaliphatic moieties bearing epoxy rings with silicon–hydrogen addition and epoxylation reactions. By incorporating flexible chain segments of (CH_2_)_n_ with different chain lengths (Figure 6a), the toughness of the cured products can be improved. Xia et al. [117] reported the use of H2004 (a hyperbranched polyester terminated with six hydroxy-branched groups) and PTMG (poly(tetramethylene ether glycol)) with linear hydroxyl groups as modifiers to enhance the toughness of UV-cured resins. The T_g_ of the prepared samples increased as the PTMG content increased, and the impact strength was dramatically improved to 10.44 J/cm^2^. The authors proposed incorporating linear PTMG as a soft segment into the cycloaliphatic epoxy resin system, which resulted in improved impact strength (seen in Figure 6b). However, the increase in toughness is often accompanied by a significant decrease in the glass transition temperature (T_g_) and phase separation of epoxy resin. In such cases, the thermal performance is relatively poor and may not meet industrial requirements [118]. To address the issue of low T_g_ and poor heat resistance in epoxy resins, Liu et al. synthesized a novel UV-curable epoxy resin modified with cholic acid and glycidyl methacrylate. The modified resin demonstrated a significant improvement in both T_g_ (144 °C) and the dielectric constant (2.51) [119]. This enhancement opens broader applications for UV-curable resins, not only in perovskite solar cell encapsulation but also in electronic product packaging.

Resin anti-aging properties can also be tailored by carefully choosing or introducing functional groups. Recent studies in the literature indicate that the aliphatic or cycloaliphatic resins are preferable to aromatic rings for modification purposes. In the field of UV curing resins, these aliphatic monomers are considered to be promising materials for enhancing the durability of systems with anti-aging properties. CY179 (3,4-Epoxycyclohexylmethyl 3,4-epoxycyclohexanecarboxylate) is the most commonly used commercial aliphatic epoxy resin in cation curing with a fast polymerization rate and low viscosity [120,121,122,123]. Gao et al. [97] proposed the use of siloxane bonds to improve UV and thermal stability. The cured resin exhibited high resistance to yellowing under 288 h of 120 °C thermal aging or UV aging, making it suitable for optical lenses and electronic sealing applications. These above-mentioned materials, already applicable to photoelectronic encapsulation (e.g., LED) and can serve as references for PSC encapsulation.

Another important parameter worth considering to avoid cracking is the removal of dissolved gas from resin. High-temperature conditions in thermal stability testing can cause gas expansion and bubble formation in UV-cured encapsulation [124], leading to delamination and failure of the encapsulation structure [109] (Figure 5a). Therefore, it is important to prioritize the degassing step before encapsulation with UV-curable resin of certain viscosity, and it is recommended to store the encapsulation adhesive in an oxygen-free environment. Additionally, during the UV curing process, it is crucial to eliminate the potential risk of vapor emitted from the UV epoxy resin for the same reason [61,125].

#### 3.2.3. UV-Curable Resin Design to Reduce the Required UV Irradiation Dose

Some photocurable adhesives require high doses of UV irradiation; therefore, it is inevitable that a perovskite solar cell will be subjected to UV damage during the encapsulation [21,22,23,24,126]. The UV-induced degradation mechanisms were summarized in Section 2 [127,128]. As a result, a lower demand for the UV irradiation dose will remarkably benefit the durability of PSC. The key to minimizing the UV irradiation requirement is to develop photoinitiators that can be excited with less harmful visible light and trigger rapid resin curing [129].

**Figure 7:**
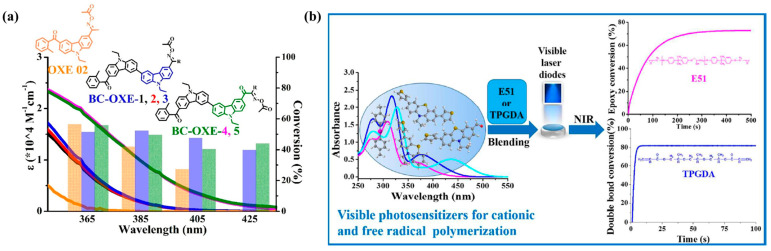
(**a**) Oxime ester type photoinitiators extend the absorption wavelength range of photoinitiators to the near-ultraviolet–visible (UV–vis) region and ensure the high efficiency of photoinduced polymerization. Reproduced with permission from Ref. [130]. Copyright 2020, Elsevier Ltd. (**b**) Thiophene-substituted phenothiazine-based photosensitizers for a radical and cationic photopolymerization reaction under soft laser diode irradiation at 405 and 455 nm. Reproduced with permission from Ref. [131]. Copyright 2016, Royal Society of Chemistry.

In a recent study by Zhou et al. [130], bicarbazole-based oxime esters, as photoinitiators, were found to exhibit outstanding performance in the photopolymerization processes, demonstrating high conversion rates and initiation rates. This highly efficient photoinitiator was proven to be effective in initializing in the field of UV-visible LED photocuring, as shown in Figure 7a. Importantly, it showed that initiation of monomer crosslinking can occur with low doses of ultraviolet radiation, even within the visible light range. The o-acyloximes exhibited superior performance compared with commercial photoinitiators when irradiated with visible LEDs at wavelengths 405 and 425 nm. Under an irradiance of 45 mW cm^−2^ and with a curing time of no more than 15 s, these compounds outperformed commercial photoinitiators, which typically require irradiation at 365 nm (light intensity between 30 and 45 mW cm^−2^). Specifically, the synthesized samples displayed enhanced double-bond conversions and polymerization rates. Chao et al. [131] proposed an approach to extend the π-conjugation of phenothiazine to enhance its visible light absorption properties (Figure 7b). They successfully synthesized thiophene-substituted phenothiazine-based photosensitizers. These photosensitizers were able to initiate the polymerization of epoxy resin under visible laser beams at 405 and 455 nm. The most efficient photopolymerization systems enabled the Suzuki coupling reaction for the cationic polymerization of E51 to achieve a conversion rate of over 80% within just 50 s of irradiation at 455 nm and under an irradiance of 30 mW cm^−2^.

The efficiency and speed of the aggregation process are influenced by various factors and are closely tied to the overall formulation. Within this formulation, the selection of monomers, pigments, additives, and other components can all exert an impact on the curing process [132]. In addition to using high-performance single photoinitiators, such as alkylamino acetophenones (AAAPs) [133,134], with efficient free radical production, multi-functional monomers usually form strong coatings much faster than mono-functional monomers due to the increased level of crosslinking. In addition to molecular design, other strategies that can increase curing speed include lowering film thickness, filling with pigments and light scattering components, introducing various hydrogen donors, and substrate reflection effects [135,136,137,138]. Multifunctional monomers and conjugated polymers present promising candidates for photovoltaic applications. For example, PPDT2FBT-Vx (x = 0, 5, 10) blending with PC71BM was found to achieve both high device efficiency and thermostability for solar cells. These UV-crosslinked PPDT2FBT-V10 solar cells displayed an initial average PCE of 5.28%, and almost no performance decline was found under heat treatment at 120 °C for 40 h [139]. Table 3 have summarized the mentioned resins in Section 3.2 including their structures and the improvement in the performance.

#### 3.2.4. Commercially Available UV-Curable Resins as Candidates for PSC Encapsulation Materials

In addition to the reported work focusing on resin optimizations, a range of commercially available UV-curable resins are widely utilized for PSC encapsulation in the literature for material-design-oriented works by absorber materials scientists. A careful choice of suitable encapsulation resin is critical for the overall performance of PSCs. For example, Reyna et al. compared two commercial resins, Threebond and Ossila, for the encapsulation of PSCs. The results demonstrated that devices using Threebond resin (3035B) showed more severe degradation in perovskite after outdoor testing for 300–500 h. However, devices using Ossila resin (E131) maintained their performance for over 1000 h under the same testing conditions. Their experiments also confirmed the harmful effects of UV radiation on the devices [95]. To provide general references for scientists focusing on perovskite layer design, commercially available UV-curable encapsulation materials are briefly reviewed in this section.

**Figure 8:**
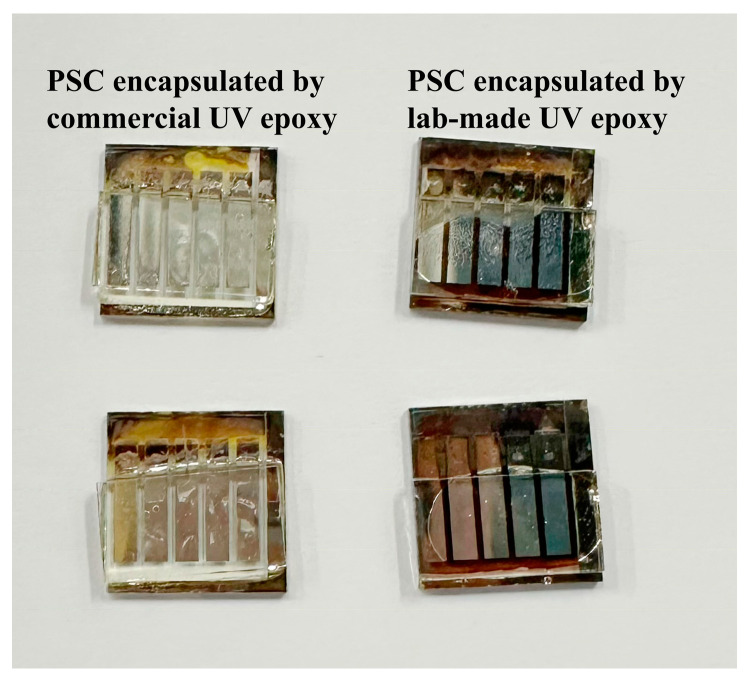
CH_3_NH_3_PbI_3_ PSC encapsulated with commercial UV-curable cycloaliphatic epoxy resins (**left**) and UV epoxy designed by the authors (**right**).

Though widely used in the literature, currently, there is no UV-curable resin specifically designed for PSCs, but there are several adhesives/encapsulants for electronic devices and other type of PV cells. The limitations of these commercial resins as PSC encapsulation materials and the corresponding properties of the most widely used commercial resins are summarized in Table 4. An example of incompatibility between commercial resins and PSCs is shown in Figure 8. When commercial resin applied as a droplet onto the deposited metal electrode of a perovskite solar cell for encapsulation, the resin can rapidly dissolve the active layer, resulting in an instant deactivation of the perovskite cell’s active layer.

## 4. Recent Advances in Encapsulation Methods Using UV-Curable Resins as PSC Encapsulation

In the literature on PSC encapsulation, UV-curable resins are usually directly used as the encapsulation material (also known as blanket cover encapsulation), or as an edge sealant for protection (known as edge sealing). In this section, we will discuss these encapsulation methods, focusing on the recent advances in UV-curable adhesives for PSC encapsulation in the literature and their specific roles within specific encapsulation structures.

### 4.1. UV-Curable Resin Used as Encapsulation Materials

**Figure 9:**
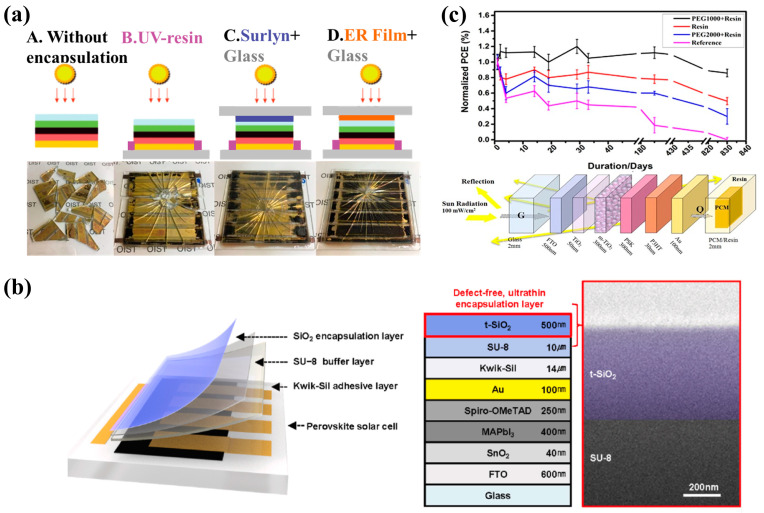
(**a**) Schematic illustration showing encapsulation methods. A, B, C, and D indicate different encapsulation strategies. The digital pictures represent different degrees of perovskite degradation under the same impact and water dripping conditions. Reproduced with permission from Ref. [142]. Copyright 2019, Nature Publishing. (**b**) Schematic showing the encapsulation layers in the PSC and illustration of the entire PSC structure with SEM image of the encapsulation layer. Reproduced with permission from Ref. [144]. Copyright 2023, Elsevier Ltd. (**c**) Schematic showing the structure and PCE of PSC with phase change material. Reproduced with permission from Ref. [127]. Copyright 2020 American Chemical Society.

UV-curable resin that is used as encapsulation materials is a straightforward strategy that benefits large-scale processing as well as provides an excellent barrier to PSC. For example, Jiang et al. [142] used a self-healable epoxy resin encapsulate film to assess lead (Pb) leakage in encapsulated modules under varying simulated natural environment. The team compared four different types of PSC modules carefully designed to evaluate degradation by measuring the Pb leakage (Figure 9a). In configuration B, the conventional UV-curing adhesive method was used, which encapsulated perovskite solar modules with 1 mm-thick glass substrates. This involved the application of UV resin on the bottom sides and edges of the modules. Module C was encapsulated with glass substrates at the bottom side similar to module B and thermo-compressed Surlyn^TM^ adhesive film on the top side. Module D was encapsulated with glass substrates at the bottom side and self-healing epoxy resin-based polymer films (with diglycidyl ether bisphenol A:n-octylamine:m-xylylene diamine  =  4:2:1) were further added at the top side. Perovskite layer degradation decreased following a sequence from A to D, with the Pb leakage rates of 34, 30, 2.6, and 0.08 mg h^−1^ m^−2^, respectively. The reason for the variation in Pb leakage primarily lies in the enhanced mechanical strength and self-healing capabilities of the epoxy resin on the coverslips. When tested in outdoor simulation environments, the rationally designed epoxy resin underwent a self-healing process, which improved the lifetime of PSC and therefore prevented the leakage of Pb.

As summarized in Section 2.1, UV-curable resin exhibits many advantages matching the requirements of an ideal PSC encapsulation material such as high transparency, low shrinkage, good adhesion, mechanical robustness, and environmental stability [61,143]; however, their performances are not perfect yet and need further improvement. In studies on the encapsulation structure design of UV-curable resin sealed PSCs, the stacking or blending of resin with other materials was proven to be effective approach. For example, Cho et al. [144] developed a three-layer encapsulation structure for PSCs with a SiO_2_ waterproof layer in which UV-curable epoxy resin (SU-8) was spin-coated and cured as a buffer layer between the SiO_2_ nanomembrane and silicone adhesive (Figure 9b). With a Young’s modulus of approximately 4 GPa, UV-curable epoxy resin provided a stable connection between the upper and lower layers with disparate Young’s moduli. This structure can be regarded as a method to reduce stress accumulation, thereby avoiding delamination and failure caused by cracking in the encapsulation layer. The overall encapsulation architecture effectively protected PSCs, allowing them to remain unaffected by external water molecules for over a month when immersed in water. Functionalizing the encapsulation layer by incorporating desired functional components into epoxy resin is another promising strategy to enhance the durability of PSCs. For example, Fumani et al. [127] reported a successful two-layer encapsulation structure composed of a resin layer with an intermediate polyethylene glycol (PEG) layer. A 500 μm thick layer of lab-made pure UV-curable epoxy resin was applied onto the PSC followed by a layer of PEG/resin blend, serving as phase-change materials for PV thermoregulation. A schematic showing the structure and the PCE of the PSC can be seen in Figure 9c. This innovative design built upon the improved waterproofing performance offered by the epoxy resin, and the incorporated phase-change components of this structure provided cooling functionality with a thickness of approximately 1 mm. Even after 830 days of aging in simulated environments, the cells maintained 80% of their initial performance.

**Figure 10:**
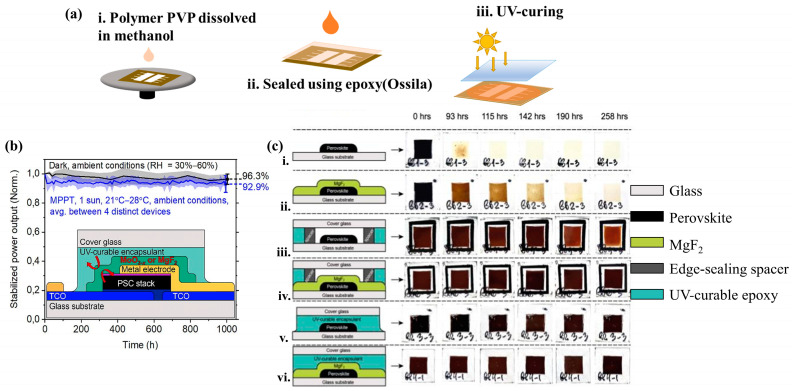
(**a**) PVP polymer intermediate layer placed between a perovskite solar cell and epoxy resin to encapsulate the PSCs. Reproduced with permission from Ref. [145] Copyright 2018, Wiley. (**b**,**c**) Inorganic capping layers, epoxy/glass sealing, and photographs of PSC encapsulated in different geometries upon a photostability test at ambient environment. (**i**) Unencapsulated perovskite film, (**ii**) perovskite film covered with evaporated MgF_2_, (**iii**) edge-encapsulated perovskite film, (**iv**) edge-encapsulated MgF_2_/perovskite film, (**v**) perovskite film encapsulated without N_2_-containing gap, (**vi**) MgF_2_/perovskite film encapsulated without N_2_-containing gap, respectively. Reproduced with permission from Ref. [146]. Copyright 2023, Elsevier Ltd.

Recent advances have also suggested that the addition of an intermediate layer is crucial to enhance the chemical compatibility between UV-curable resin and perovskite crystal because direct contact between the UV-curable adhesive, and the PSC can result in the detrimental effects of the vapor emitted from the ultraviolet epoxy resin during the UV curing process [61,125]. For example, Stringer et al. [145] used a solution-processable polyvinylpyrrolidone (PVP) polymer intermediate layer placed between a perovskite solar cell and epoxy resin to encapsulate the PSCs. As illustrated in Figure 10a, the polymer PVP dissolved in methanol spin-cast onto the device prevented perovskite degradation by UV-curable epoxies. PSC demonstrated 80% of its initial performance after 1500 h of continuous illumination at 1500 W Xenon lamp irradiation, 42 ± 3 °C, and 38 ± 6% RH condition. Belich et al. [146] demonstrated the significance of placing encapsulation material directly on top of the active layer of PSCs without leaving any gas gaps between the material and the glass substrate. Comparing the positioning of UV-curable resin encapsulation, it was observed that the presence of resin at the edge of the cell resulted in the formation of nitrogen gas-filled voids within the cell, leading to noticeable degradation in the perovskite layer. On the contrary, when the perovskite layer was completely surrounded, this issue was avoided (Figure 10c). Similarly, they used a vacuum thermal evaporation method to first deposit an inorganic cover layer (MgF_2_) and then applied the encapsulation resin (5–7 μL of epoxy from FunToDo) to prevent direct contact between the perovskite active layer and the UV-curable resin during curing. In this structure (Figure 10b), UV-curable resin acted as an additional moisture barrier and adhesive for the coverslip. As a result, the degradation of PSCs can be significantly reduced under atmospheric conditions and irradiation.

### 4.2. UV-Curable Resin Used as an Edge Sealant

**Figure 11:**
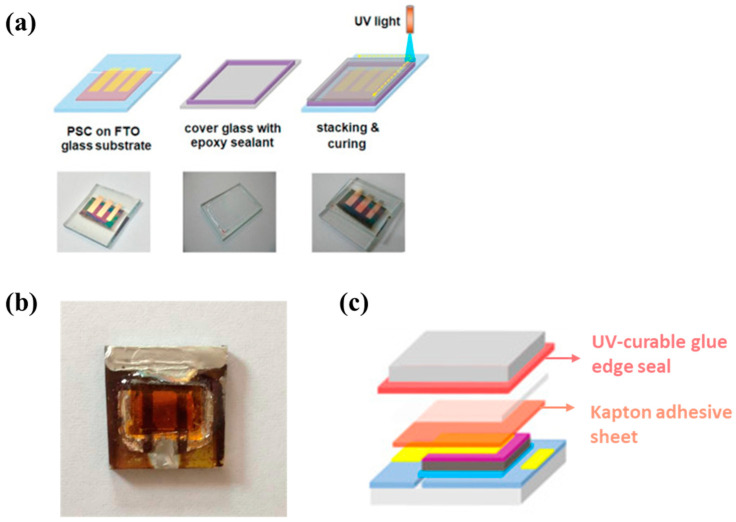
(**a**) The encapsulation of a PSC device using Vitralit as an edge sealant. Reproduced with permission from Ref. [45]. Copyright 2019, Elsevier Ltd. (**b**) Edge-encapsulated device. Reproduced with permission from Ref. [73]. Copyright 2015 American Chemical Society. (**c**) Schematic showing the sealing strategy with a Kapton adhesive sheet and UV-curable edge seal. Reproduced with permission from Ref. [71]. Copyright 2016, Elsevier Ltd.

The UV-curable resin used as an edge-encapsulated device allows ultraviolet light to be selectively irradiated at the edge, which greatly alleviates the damage caused by ultraviolet incidents to the perovskite active layer. In Ramasamy et al.’s study [45], an epoxy resin was used to seal edges around the device, which formed a layered structure that was selectively irradiated at the edges using UV light (Figure 11a). The UV-curing epoxy used as edge sealant was produced from Vitralit. This PSC retained 80% of its original performance after 70 days when the device was exposed to 30 °C temperature and 50% humidity. In PSC studies, similar structures that solely use UV-curable adhesive for edge encapsulation prove to be a convenient encapsulation method, as depicted in Figure 11b [147]. Another simplified approach involves the utilization of cavity glass with a direct application of UV-curable epoxy as the edge sealant. [148,149]. Shi et al. first conducted “blanket encapsulation” on cells and then suggested that UV-curable epoxy resin might not exhibit inert behavior toward the materials of the cell [61]. The results showed that using an edge encapsulation structure with only FTO feed-through and no Au feed-through could result in a relatively smaller (8%) loss in PCE after 30 thermal cycles.

Similar to using UV-curable resins directly as encapsulation materials, combining UV-curable resins used as PSC edge sealant with other encapsulants was proven to provide high stability for PSCs. By adding another functional layer or barrier layer, a stronger capacity to block water vapor and oxygen can be achieved. Moreover, this addition can prevent direct contact between the epoxy resin and the perovskite active layer. Matteocci et al. [71] comprehensively evaluated the performance of different types of encapsulations for high-stability PSCs (Figure 11c). They proved that a combination of thermal film, UV-curable and photocurable adhesives, Kapton polyimide adhesive, and UV-curable edge sealing showed an outstanding stability-enhancing function for PSCs even in harsher environments.

**Figure 12:**
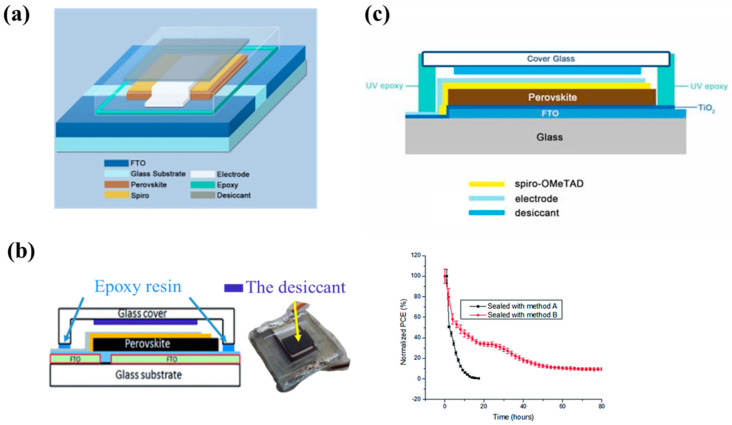
(**a**) Diagram showing an encapsulated cell. Reproduced with permission from Ref. [150] Copyright 2016, Wiley. (**b**) Schematic cross-section showing the method B-sealed perovskite solar cells along with the corresponding PCE of two sealing methods (method A: epoxy resin, method B: epoxy resin with desiccant). Reproduced with permission from Ref. [125]. Copyright 2015, Royal Society of Chemistry (**c**) Schematic diagram showing an encapsulated solar cell. Reproduced with permission from Ref. [151] Copyright 2016, Wiley.

Another representative approach is the combination of a desiccant layer and UV-curable sealant. In a recent work, Dong et al. [150] used an electron beam to deposit a 50 nm thick SiO_2_ layer on the electrode then protected it with a coverslip and a piece of desiccant, a diagram showing the encapsulated cell shown in Figure 12a. The edge of PSC was sealed with epoxy glue under the condition of 1 min of UV illumination for UV-curable epoxy followed by annealing at 80 °C for 10 min under UV illumination for thermally curation. Overall, 85% initial efficiency was retained after 144 h under constant light irradiation conditions of 65% relative humidity (RH) and 85 °C, and 90% PCE was retained after 432 h of testing under outdoor conditions. Han et al. [125] compared the encapsulation of MAPbI_3_ devices using epoxy resin edge sealants with and without desiccants (Figure 12b). The results demonstrated that when encapsulation sealants and desiccants were both used, the devices exhibited a longer lifetime at a higher temperature (85 °C) and high humidity (80% RH), as indicated by red line in the PCE graph in Figure 12b. Liu et al. [151] introduced UV-curable adhesives for edge sealing and a 180 μm desiccant from Dynic Corporation desiccants into the encapsulation structure and tested the moisture and high-temperature tolerance of the perovskite film (Figure 12c). After 10 h of illumination and 14 h of darkness (maintaining 65% RH), the PSC cell retained 90% of its initial efficiency. Under constant illumination for 36 h under the conditions of thermal testing, the cell maintained 80% of its initial efficiency. The structure combining a desiccant and a UV-curing edge adhesive ensures an extremely low local water vapor concentration inside the PSC even under extreme weather conditions. Although this structure design achieved many successes, as reported in the literature, the presence of gases (typically, nitrogen/argon in glovebox) in the desiccant-embedded cover glass design can result in undesired outcomes like bubble formation and delamination in the epoxy resin, which can adversely affect the lifespan of the device. Therefore, combining glass with grooves and desiccants may need further delicate optimization for PSC encapsulation [109].

**Figure 13:**
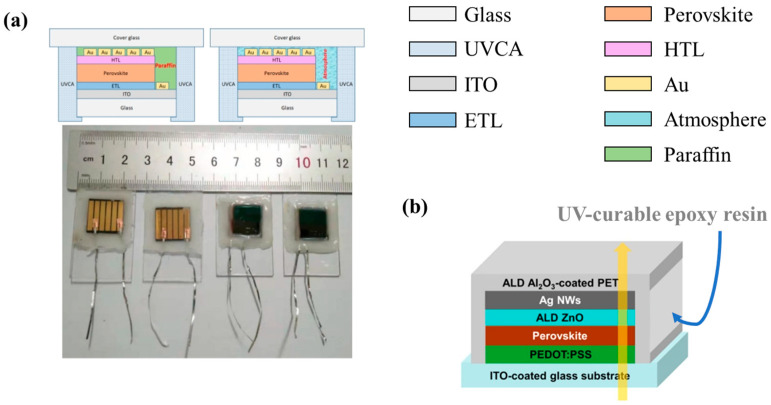
(**a**) A schematic showing an encapsulation structure of UV-curable resin with and without paraffin and a two-sided photo-image of device. Reproduced with permission from Ref. [68] Copyright 2020, Wiley. (**b**) ALD Al_2_O_3_ film coating on a PET substrate at 100 °C as the top encapsulation layer with edge sealing achieved using a UV-curable epoxy resin. Reproduced with permission from Ref. [80] Copyright 2015 American Chemical Society.

Combining functional layers that reduce the contents of residual oxygen with UV-curable sealants has also proven to be effective for enhancing the stability of PSCs. For instance, Ma et al. [68] used non-polar paraffin as an encapsulation material together with UV-cured adhesive edge sealant (Figure 13a). The non-polar nature of paraffin ensures its high efficacy for removing oxygen and moisture during encapsulation. The reported PSC achieved an excellent long-term stability that maintained 80% of the initial value after 1000 h in natural environments. Surlyn^TM^ film was also reported to act excellently along with epoxy glue as UV-curable edge sealants for PV cells [152,153]. Grancini et al. [154] prepared a PSC with an active area of 50 cm^2^, which showed remarkable stable efficiency for one year when kept under sun conditions (55 °C) using Surlyn^TM^ encapsulation with an additional ring of epoxy resin as a second layer of protection.

UV-curable resins as edge sealants also work perfectly with inorganic encapsulation materials. Chang et al. [80], for instance, utilized atomic layer deposition (ALD) to take advantage of ALD’s unique capabilities in forming excellent conformal films with high uniformity over large area substrates. They applied a 50 nm ALD Al_2_O_3_ film coating on a PET substrate at 100 °C as the top encapsulation layer, while the edge sealing was achieved using a UV-curable epoxy resin from Electron-lite Corp. After being kept in ambient atmosphere at 30 °C and 65% RH for 40 days, this device (structure shown in Figure 13b) retained over 95% of its initial PCE. The remarkably high PCE not only stemmed from the application of ALD ZnO film as an additional cathode buffer layer in PSCs but also from the excellent gas barrier properties provided by the epoxy resin-Al_2_O_3_ encapsulation structure.

### 4.3. UV-Curable Resin Used in Other Structures

In addition to serving only as encapsulation materials, UV-cured materials were reported to exhibit additional functionality in PSC encapsulation. Several studies demonstrated their utilization in various aspects of PSC encapsulation, showcasing their potential for enhancing performance and functionality beyond basic encapsulation properties.

**Figure 14:**
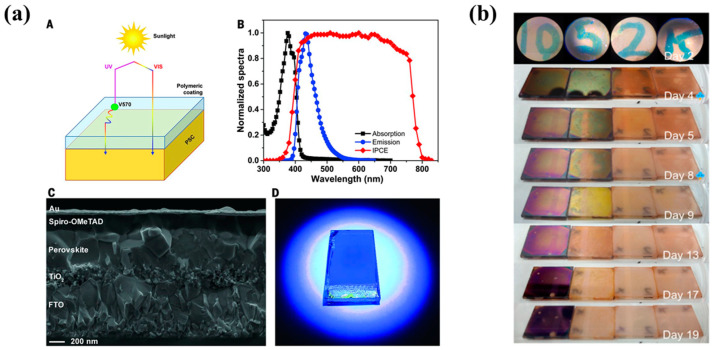
(**a**) (**A**) Scheme showing the UV-coating operating principle. (**B**) Normalized absorption and emission spectra of V570-doped UV coating compared with the IPCE response of a PSC device under study. (**C**) Cross-sectional field-emission scanning electron microscopy image of a PSC device before coating deposition. (**D**) Digital photograph showing a PSC bearing a UV coating when exposed to UV light. Reproduced with permission from Ref. [107] Copyright 2016, Science. (**b**) Stability performance. Photos of samples with different amounts of UV-gel in HTM. Reproduced with permission from Ref. [62]. Copyright 2016, Elsevier Ltd.

The use of fluorinated polymers can enhance the waterproofing performance of encapsulation materials. Bella et al. [107] utilized a UV-curable fluoropolymeric binder, which was a mixture of a chloro-trifluoro-ethylene vinyl ether resin (Lumiflon LF-910LM, Asahi Glass Corporation, Tokyo, Japan) and a difunctional methacrylic perfluoropolyether oligomer (Fluorolink MD700, Solvay Specialty Polymers, Brussels, Belgium), to form a highly hydrophobic barrier on the backside of cells, providing protection against environmental humidity. By incorporating a fluorescent organic dye (Lumogen F Violet 570 by BASF) into this coating, UV irradiation was blocked on the light incident side (front side) of the PSC, converting UV light into visible light and increasing the photocurrent by 6% (details shown in Figure 14a). These cells with UV-curable fluoropolymeric binder on the outer face of FTO (F-doped tin oxide) glass substrate and on top of the gold back contact acting as the encapsulation material successfully passed outdoor stability and aging tests.

Li et al. [62] presented a novel UV curing recipe involving ethoxylated trimethylolpropane triacrylate (ETPTA) and 2-hydroxy-2-methyl-1-phenyl-1-propanone with UV-cured ETPTA used as an additive rather than an encapsulation material (Figure 14b). With cross-linked frames that prevent water from passing through the device, introducing a suitable amount of UV-gel in the perovskite precursor solution and hole transport material solution during device fabrication helped to enhance perovskite crystallization, minimize moisture diffusion in the hole transport layer, and subsequently mitigate degradation of PSCs.

### 4.4. Comparison between “Blanket-Cover” and “Edge-Sealant” Structures

**Figure 15:**
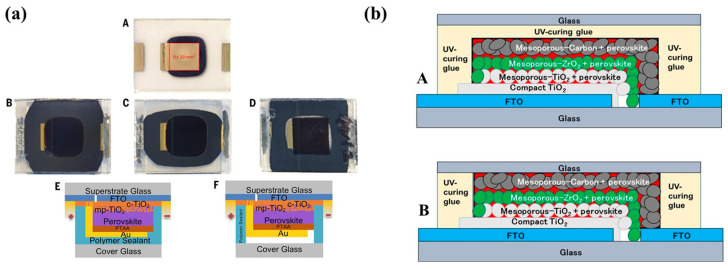
(**a**) (**A**) A metal-side view of PSC before packaging. (**B**–**D**) Superstrate side views of PSC after PIB-based blanket encapsulation. (**E**,**F**) Illustrations showing side views of two encapsulation structures. Reproduced with permission from Ref. [60]. Copyright 2020, AAAS. (**b**) Diagram showing a fabricated three-layer PSC device: (**A**) over-sealed and (**B**) side-sealed cell. Reproduced with permission from Ref. [155] Copyright 2016, Wiley.

In summary, in the literature, the main roles of UV-curable resins are “blanket-cover” encapsulants or edge sealants as a part of PSCs. However, the superiority of these two methods is case-specific. For example, Shi et al. [60] utilized polyisobutylene (PIB) and polyolefin (PO) materials to form a blanket-cover structure using a cost-effective and straightforward packaging protocol. The thermal cycling tests based on the gas chromatography-mass spectrometry (GC-MS) analysis demonstrated that both the “blanket-cover” encapsulation method and edge sealing are beneficial in preserving the stability of PSCs; an illustration of the structure is shown in Figure 15a. However, the “blanket-cover” approach exhibited superior stability under extreme temperature and humidity conditions due to its ability to provide a “tight-pressure” environment, which suppressed the rapid decomposition reaction of PSCs and prevented the release of volatile decomposition by-products. Additionally, the study suggested that wider encapsulation edges provided better barrier performance compared with narrower edges. Baranwal et al. [155] found that the stability of a three-layer printable hole transport material (HTM)-free CH_3_NH_3_PbI_3_ devices was drastically affected by the position of UV-curing glue (Threebond, 3035B). With the UV-cured epoxy providing the same barrier performance against external moisture, it was observed that the side-sealed cells demonstrated better stability in terms of PCE compared with the over-sealed cell structures on the carbon layer during thermal stability testing (the side view of PSC could be found in Figure 15b). The authors suggested that the edge sealing of epoxy minimized the occurrence of voids, thereby no gas would be trapped inside the encapsulated PSCs.

## 5. Summary and Perspective

In summary, PSCs have attracted tremendous attention due to their high efficiency and low cost. However, stability issues of PSCs induced by lattice structures, defects, heat, oxygen, moisture, and illumination, hinder the practical application and commercialization of PSCs. UV-curable resins, a common encapsulation material for PSCs, exhibit the advantages of a short curing time and good adhesion properties and can form a strong and uniform encapsulation layer that provides effective protection against environmental factors. UV-curable encapsulating materials with tailored molecular structure and encapsulation methods have shown low WVTR and OTR values, low elastic modulus and shrinkage, high mechanical strength, and great chemical stability beneficial for the long-term stability of PSCs. More importantly, the encapsulation condition required by UV-curable resins is more tolerable to PSCs compared with the high temperature and pressure required for other encapsulation films.

However, the weather resistance and anti-aging properties of UV-curable encapsulating adhesives still require further improvements, specifically for PSC encapsulation. Recent research indicates that epoxy resins used in UV-curable encapsulation release gases that damage perovskite active layers. The challenges in long-term UV light exposure that induce yellowing, cracking, and ultimately failure of encapsulation have not been thoroughly addressed [156]. Additionally, innovative design of encapsulation architecture based on UV-curable adhesives needs to be developed accordingly. Based on the analysis of the encapsulation architecture related to PSCs, future works are encouraged to focus on improving the ratio of UV-curable adhesives and finding chemically and thermally compatible additional functional layers along with UV-curable resins for PSC encapsulation. The goals are as follows:

1. To prevent the encapsulating adhesive from reacting with the core layers of the cells;
2. To enhance the toughness of the UV-curable encapsulating adhesive to adapt to shrinkage deformation in the PSC encapsulation cover plate;
3. To meet the application requirements in the encapsulation process of future flexible solar cells [157].

Another issue with UV-curable epoxy that is worthy of attention is the necessity of moisture/gas outgassing. During the curing process, the epoxy can release trapped moisture/gas, which could potentially degrade the performance of solar cells. A degassing process or the use of desiccant material is required when using epoxy as an encapsulant. This additional step makes it more labor-intensive and increases the likelihood of errors. Furthermore, the need for such additional steps could also escalate the overall costs of solar cell production. UV-curable epoxy is typically available in liquid form and composed of monomers, initiators, stabilizers, and trace amounts of moisture and oxygen. These constituent materials must not react with perovskite layers in the solar cells. However, ensuring such non-reactivity has proven to be challenging, which makes the encapsulation process more complex. This demands meticulous selection and careful handling of the epoxy, potentially making the process time-consuming and intricate. For PSC production on the industrial scale, optimizing the scaling-up process including improving reproducibility, reducing manufacturing costs, and adapting to the needs of large-scale processing equipment are also critical to bringing PSCs from lab to practical application. 

## Figures and Tables

**Table 1:** Specifications for OPV encapsulant materials. Reproduced with permission from Ref. [39]. Copyrights 2013, Elsevier Ltd.

Characteristics	Specifications
WVTR (water vapor transmission rate)	10^−4^–10^−6^ g m^−2^ day^−1^
OTR (oxygen transmission rate)	10^−3^–10^−5^ cm^3^ m^−2^ day^−1^ atm^−1^
Glass transition temperature (T_g_)	<−40 °C (during winter in deserts)
Total light transmission	>90% of incident light
Water absorption	<0.5 wt% (20 °C/100% RH)
Tensile modulus	<20.7 MPa (<3000 psi) at 25 °C
UV absorption degradation	None (>350 nm)
Total hemispherical light transmission over the wavelength range from 400 nm to 1100 nm	>90% of incident light
Chemical inertness	No reaction (with embedded Cu coupons at 90 °C)
Resistance to thermal oxidation	Stable (up to 85 °C)
Hydrolysis	None (80 °C, 100% RH)
Mechanical creep	None (90 °C)
Hazing or clouding	None (80 °C, 100% RH)

**Table 2:** Overview of the characteristics of encapsulation materials.

Encapsulation Materials	WVTR (g/m^2^/Day)	Processing Time and Pressure	Processing Temp.	Refs.
EVA	~28.0	Lamination 15–30 min at vacuum 100 kPa	~140–~150 °C	[66,67]
POE	~3.8	Lamination 15–30 min at vacuum 100 kPa	~90–~130 °C	[66]
PET	1 × 10^−2^ to 1 × 10^−3^	Used as substrate and applied to the PSC with UV-curable epoxy	RT	[80,81]
PIB	1 × 10^−2^ to 1 × 10^−3^	Hot-pressed with a background pressure of 300–400 mTorr for ~10 min	RT-~160 °C	[61,66]
PMMA	~55.2	Solution spin-coating and 5 min on hot plate	~80 °C	[63]
EVOH	~4.72 × 10^−2^	Precursor solution heated on a hot plate to form EVOH film and adhesive with UV-curable epoxy	~70 °C	[64]
TPU	N/A	Spray-painted PU resin or vacuum 100 kPa crosslink at RT for ~24 h	RT-~110 °C	[66,82]
UV-cured resin	10^2^ to 10^−1^	UV light 30–45 mW cm^−2^ illuminate 10 s–10 min	RT	[45,61,71]

**Table 3:** Category of resins described in Section 3.2.

Resin Composition	Structure	Pros	Ref
Transparent cycloaliphatic epoxy–silicone	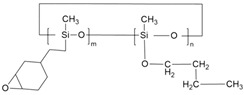	Higher transmittances, lower water absorptions, better UV/thermal resistance and thermal stabilities	[97]
Transparent polyurethane acrylate–mesoporous silica nanoparticles composites	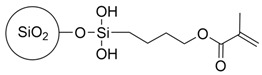	High transparency and low haze, low moisture permeability and high adhesive force	[98]
Ethyleneglycoldimethacrylate-bridged polysilsesquioxane	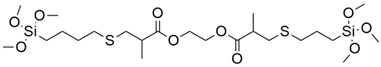	Better water vapor barrier property and adhesion property	[103]
Cycloaliphatic epoxy-oligosiloxane	See Figure 4b	High optical transparency, low permeability	[94]
Aliphatic hydrophobic backbone mixed 2-(perfluorohexyl) ethyl methacrylate	Mixture of CN991 with 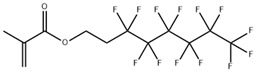	Lower surface energy and a better resistance to corrosion.	[105]
2-isocyanatoethyl methacrylate and 2-isocyanatoethyl methacrylate	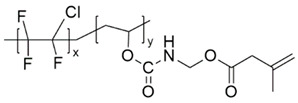 with EuD4TEA	Down-shifting material to convert UV photons into valuable visible light and high hydrophobic	[106]
Epoxy resins–cycloaliphatic moieties bearing epoxy rings	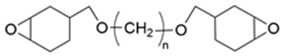	Improved toughness	[116]
Cycloaliphatic epoxy-oligosiloxane-hyperbranched polyester terminated—six hydroxy-branched groups and poly(tetramethylene ether glycol)	See Figure 6b	Enhance the toughness and impact strength	[117]
Epoxy cresol novolac modified with cholic acid and glycidyl methacrylate	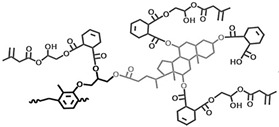	High glass transition temperature and low dielectric constant	[119]

**Table 4:** Summary of commercial encapsulation UV-curable resin.

Manufacturer	Model	Product Type	T_g_	Ingredients	WVTR (g/m^2^/Day)	Refs. of Implementation
Three Bond Holdings Co., Ltd. (Tokyo, Japan)	Threebond 3035B	Acrylic resin	N/A	Silica 45–55 wt%; acrylate monomers, acrylate oligomers, photoinitiators, additives 50–60 wt%	97 (@85 °C, 85%RH)	[68,71,95,140]
Ossila (Sheffield, UK)	Ossila E131	Carboxylic acids epoxy resin	138 °C	Epoxy resin 96 wt%, photoinitiators > 3 wt%, photostabilizers > 3 wt%	N/A	[95,96]
Nagase ChemeteX (Osaka, Japan)	XNR5516ZD	Epoxy	102 °C	Epoxy resin 60–70 wt%; inorganic filler 30–40 wt%	20 (@60 °C, 90%RH)	[61,92,141]
Electron-lite Corp. (Bethel, MN, USA)	ELC-2500	Epoxy	N/A	N/A	N/A	[80]
Asahi Glass Corporation (Tokyo, Japan)	Lumiflon LF-910LM	Fluoropolymers	37 °C	Fluoroethylene-alkylvinyl ether alternative copolymer	N/A	[107]
Panacol (Frankfurt, Germany)	Vitralit	Epoxy	>150 °C	Epoxy 80 wt%; SiO_2_ 20 wt%	N/A	[45]
Norland Products Inc. (Jamesburg, NJ, USA)	NOA 63	Urethane related resin	N/A	Mercapto esters 50–75 wt%; Triallyl isocyanuarte 15–40 wt%	N/A	[64,81]

## Data Availability

Not applicable.
